# Supplementary material for: Agricultural water allocation with climate change based on gray wolf optimization in a semi-arid region of China
Source: PeerJ. 2023 Jan 3;11:e14577. doi: 10.7717/peerj.14577 (PMC9817936; doi:10.7717/peerj.14577)
Supplement: Supplemental Information 1 [file peerj-11-14577-s001.docx]

Supplementary

Table 1 Calibration and verification of SDSM of all weather stations in the basin.

| Calibration | Highest temperature | | Lowest temperature | | Annual rainfall | | Daily relative humidity | | Annual sunlight | |
| --- | --- | --- | --- | --- | --- | --- | --- | --- | --- | --- |
|  | R2 | RMSE (°C) | R2 | RMSE (°C) | R2 | RMSE (°C) | R2 | RMSE (°C) | R2 | RMSE (°C) |
| Aksu weather station | 0.947 | 2.748 | 0.935 | 2.762 | 0.308 | 1.419 | 0.586 | 9.565 | 0.263 | 2.865 |
| Keping weather station | 0.952 | 2.8 | 0.938 | 3.347 | 0.317 | 1.402 | 0.622 | 9.838 | 0.274 | 2.782 |
| Alar weather station | 0.957 | 2.562 | 0.949 | 2.718 | 0.332 | 1.041 | 0.713 | 11.946 | 0.293 | 2.794 |
| Akqi weather station | 0.906 | 5.661 | 0.895 | 3.334 | 0.315 | 2.546 | 0.699 | 12.016 | 0.288 | 2.406 |
| Validation | Highest temperature | | Lowest temperature | | Rainfall | | Daily relative humidity | | Annual sunlight | |
|  | R2 | RMSE (°C) | R2 | RMSE (°C) | R2 | RMSE (°C) | R2 | RMSE (°C) | R2 | RMSE (°C) |
| Aksu weather station | 0.982 | 2.923 | 0.938 | 2.865 | 0.272 | 1.496 | 0.656 | 12.35 | 0.262 | 2.956 |
| Keping weather station | 0.984 | 2.915 | 0.944 | 2.915 | 0.201 | 1.633 | 0.677 | 12.13 | 0.257 | 2.862 |
| Alar weather station | 0.984 | 2.823 | 0.941 | 2.956 | 0.225 | 1.558 | 0.667 | 12.814 | 0.272 | 2.957 |
| Akqi weather station | 0.961 | 6.056 | 0.887 | 3.391 | 0.217 | 2.938 | 0.655 | 11.915 | 0.235 | 2.536 |

Table 2 Water resource allocation of various land use types throughout the basin in Actual and Recent.

| Water demand structure（%） | Farmland | Grassland | Forest land | Construction land | Water area | Unused | Total | Total water  （${10}^{8}m^{3}$） |
| --- | --- | --- | --- | --- | --- | --- | --- | --- |
| 2018 | PL | 0.1 | 9.6 | 2.7 | 12.5 | 0.0 | 100.0 | 51.87 |
| 2020 | 68.5 | 0.2 | 10.9 | 3.7 | 16.7 | 0.0 | 100.0 | 47.19 |

Table 3 Water resource allocation of various land use types throughout the basin in RCP4.5.

| Water demand structure（%） | Farmland | Grassland | Forest land | Construction land | Water area | Unused | Total | Total water  （${10}^{8}m^{3}$） |
| --- | --- | --- | --- | --- | --- | --- | --- | --- |
| 2035 | 65.0 | 0.2 | 11.3 | 6.2 | 17.4 | 0.0 | 100.0 | 50.47 |
| 2050 | 59.5 | 0.2 | 12.8 | 6.3 | 21.3 | 0.0 | 100.0 | 50.37 |

Table 4 Area allocation of various land use types throughout the basin in Actual and Recent.

| Area ($\mathrm{km}^{2}$) | Farmland | Grassland | Forest land | Construction land | Water area | Unused | Total |
| --- | --- | --- | --- | --- | --- | --- | --- |
| 2018 | 4327.7 | 15,136.6 | 1395.2 | 928.1 | 639.3 | 13661.6 | 36,088.5 |
| 2020 | 3700.5 | 15,305.9 | 1451.6 | 1190.5 | 778.4 | 13661.6 | 36,088.5 |

Table 5 Area allocation of various land use types throughout the basin in RCP4.5.

| Area ($\mathrm{km}^{2}$) | Farmland | Grassland | Forest land | Construction land | Water area | Unused | Total |
| --- | --- | --- | --- | --- | --- | --- | --- |
| 2035 | 3650.0 | 15188.8 | 1459.9 | 1300.7 | 827.4 | 13661.6 | 36088.5 |
| 2050 | 3702.7 | 14432.0 | 1751.9 | 1547.4 | 992.9 | 13661.6 | 36088.5 |

Table 6 Water resource allocation of various land use types throughout the basin in RCP8.5.

| Water demand structure(%) | Farmland | Grassland | Forest land | Construction land | Water area | Unused | Total | Total water(10^8^m^3^) |
| --- | --- | --- | --- | --- | --- | --- | --- | --- |
| 2035 | 66.2 | 0.2 | 11.0 | 6.0 | 16.6 | 0.0 | 100.0 | 51.17 |
| 2050 | 60.5 | 0.2 | 12.7 | 6.2 | 20.5 | 0.0 | 100.0 | 50.66 |

Table 7 Area allocation of various land use types throughout the basin in RCP8.5.

| Area ($\mathrm{km}^{2}$) | Farmland | Grassland | Forest land | Construction land | Water area | Unused | Total |
| --- | --- | --- | --- | --- | --- | --- | --- |
| 2035 | 3701.0 | 15,172.2 | 1482.0 | 1278.8 | 792.9 | 13,661.6 | 36,088.5 |
| 2050 | 3709.4 | 14,470.7 | 1778.4 | 1523.3 | 945.1 | 13,661.6 | 36,088.5 |

Table 8 Allocation of land use types in each county in the basin in Actual and Recent.

| Actual  2018(km^2^) | Farmland | Grassland | Forest land | Construction land | Water area | Unused | Total |
| --- | --- | --- | --- | --- | --- | --- | --- |
| Wushi | 535.7 | 6134.5 | 390.1 | 95.1 | 136.2 | 1401.6 | 8693.2 |
| Wensu | 848.2 | 5764.8 | 214.8 | 146.3 | 47.0 | 3966.6 | 10,987.7 |
| Keping | 67.6 | 2147.2 | 290.8 | 27.3 | 21.2 | 5107.2 | 7661.3 |
| Awati | 699.6 | 508.6 | 229.4 | 129.1 | 286.4 | 75.6 | 1928.7 |
| Aksu | 1128.1 | 498.8 | 148.9 | 365.2 | 70.5 | 2535.9 | 4747.4 |
| Alar | 1048.5 | 82.7 | 121.2 | 165.2 | 78.0 | 574.7 | 2070.2 |
| Total | 4327.7 | 15,136.6 | 1395.2 | 928.1 | 639.3 | 13,661.6 | 36,088.5 |
| Recent 2020 (km^2^) | Farmland | Grassland | Forest land | Construction land | Water area | Unused | Total |
| Wushi | 696.4 | 5851.1 | 443.4 | 123.6 | 177.1 | 1401.6 | 8693.2 |
| Wensu | 672.4 | 5966.6 | 157.0 | 177.8 | 47.3 | 3966.6 | 10,987.7 |
| Keping | 47.3 | 2241.5 | 205.8 | 32.0 | 27.6 | 5107.2 | 7661.3 |
| Awati | 489.7 | 540.6 | 298.3 | 167.8 | 356.7 | 75.6 | 1928.7 |
| Aksu | 813.2 | 638.4 | 193.6 | 474.7 | 91.7 | 2535.9 | 4747.4 |
| Alar | 981.5 | 67.7 | 153.6 | 214.6 | 78.1 | 574.7 | 2070.2 |
| Total | 3700.5 | 15,305.9 | 1451.6 | 1190.5 | 778.4 | 13661.6 | 36,088.5 |

Table 9 Allocation of land use types in each county in the basin in RCP4.5.

| RCP4.5 2035 (km^2^) | Farmland | Grassland | Forest land | Construction land | Water area | Unused | Total |
| --- | --- | --- | --- | --- | --- | --- | --- |
| Wushi | 835.7 | 5790.8 | 362.1 | 125.0 | 177.9 | 1401.6 | 8693.2 |
| Wensu | 717.3 | 5933.5 | 134.0 | 180.8 | 55.5 | 3966.6 | 10,987.7 |
| Keping | 56.8 | 2178.9 | 246.9 | 38.4 | 33.1 | 5107.2 | 7661.3 |
| Awati | 498.8 | 505.8 | 300.3 | 190.9 | 357.3 | 75.6 | 1928.7 |
| Aksu | 662.7 | 698.5 | 232.3 | 508.1 | 110.0 | 2535.9 | 4747.4 |
| Alar | 878.7 | 81.3 | 184.3 | 257.5 | 93.7 | 574.7 | 2070.2 |
| Total | 3650.0 | 15,188.8 | 1459.9 | 1300.7 | 827.4 | 13,661.6 | 36,088.5 |
| RCP4.5 2050 (km^2^) | Farmland | Grassland | Forest land | Construction land | Water area | Unused | Total |
| Wushi | 1002.8 | 5490.7 | 434.5 | 150.0 | 213.5 | 1401.6 | 8693.2 |
| Wensu | 860.8 | 5716.0 | 160.8 | 216.9 | 66.6 | 3966.6 | 10,987.7 |
| Keping | 68.1 | 2103.9 | 296.3 | 46.1 | 39.7 | 5107.2 | 7661.3 |
| Awati | 411.0 | 424.0 | 360.3 | 229.1 | 428.7 | 75.6 | 1928.7 |
| Aksu | 591.1 | 600.0 | 278.7 | 609.7 | 132.0 | 2535.9 | 4747.4 |
| Alar | 768.8 | 97.5 | 221.2 | 295.5 | 112.4 | 574.7 | 2070.2 |
| Total | 3702.7 | 14,432.0 | 1751.9 | 1547.4 | 992.9 | 13,661.6 | 36,088.5 |

Table 10 Allocation of land use types in each county in the basin in RCP8.5.

| RCP8.5 2035 (km^2^) | Farmland | Grassland | Forest land | Construction land | Water area | Unused | Total |
| --- | --- | --- | --- | --- | --- | --- | --- |
| Wushi | 835.7 | 5610.6 | 532.1 | 136.1 | 177.1 | 1401.6 | 8693.2 |
| Wensu | 701.0 | 5944.3 | 136.4 | 191.8 | 47.6 | 3966.6 | 10,987.7 |
| Keping | 39.1 | 2252.9 | 196.9 | 37.3 | 27.9 | 5107.2 | 7661.3 |
| Awati | 396.3 | 612.9 | 304.3 | 182.1 | 357.5 | 75.6 | 1928.7 |
| Aksu | 736.5 | 697.4 | 175.9 | 508.3 | 93.4 | 2535.9 | 4747.4 |
| Alar | 992.3 | 54.2 | 136.3 | 223.2 | 89.5 | 574.7 | 2070.2 |
| Total | 3701.0 | 15,172.2 | 1482.0 | 1278.8 | 792.9 | 13,661.6 | 36,088.5 |
| RCP8.5 2050 (km^2^) | Farmland | Grassland | Forest land | Construction land | Water area | Unused | Total |
| Wushi | 1002.8 | 5274.4 | 638.5 | 163.3 | 212.5 | 1401.6 | 8693.2 |
| Wensu | 841.2 | 5728.9 | 163.7 | 230.1 | 57.1 | 3966.6 | 10,987.7 |
| Keping | 46.9 | 2192.7 | 236.3 | 44.8 | 33.5 | 5107.2 | 7661.3 |
| Awati | 322.3 | 524.5 | 365.2 | 218.6 | 422.6 | 75.6 | 1928.7 |
| Aksu | 593.2 | 685.2 | 211.1 | 609.9 | 112.0 | 2535.9 | 4747.4 |
| Alar | 903.0 | 65.0 | 163.6 | 256.6 | 107.3 | 574.7 | 2070.2 |
| Total | 3709.4 | 14,470.7 | 1778.4 | 1523.3 | 945.1 | 13,661.6 | 36,088.5 |

| 2020 | 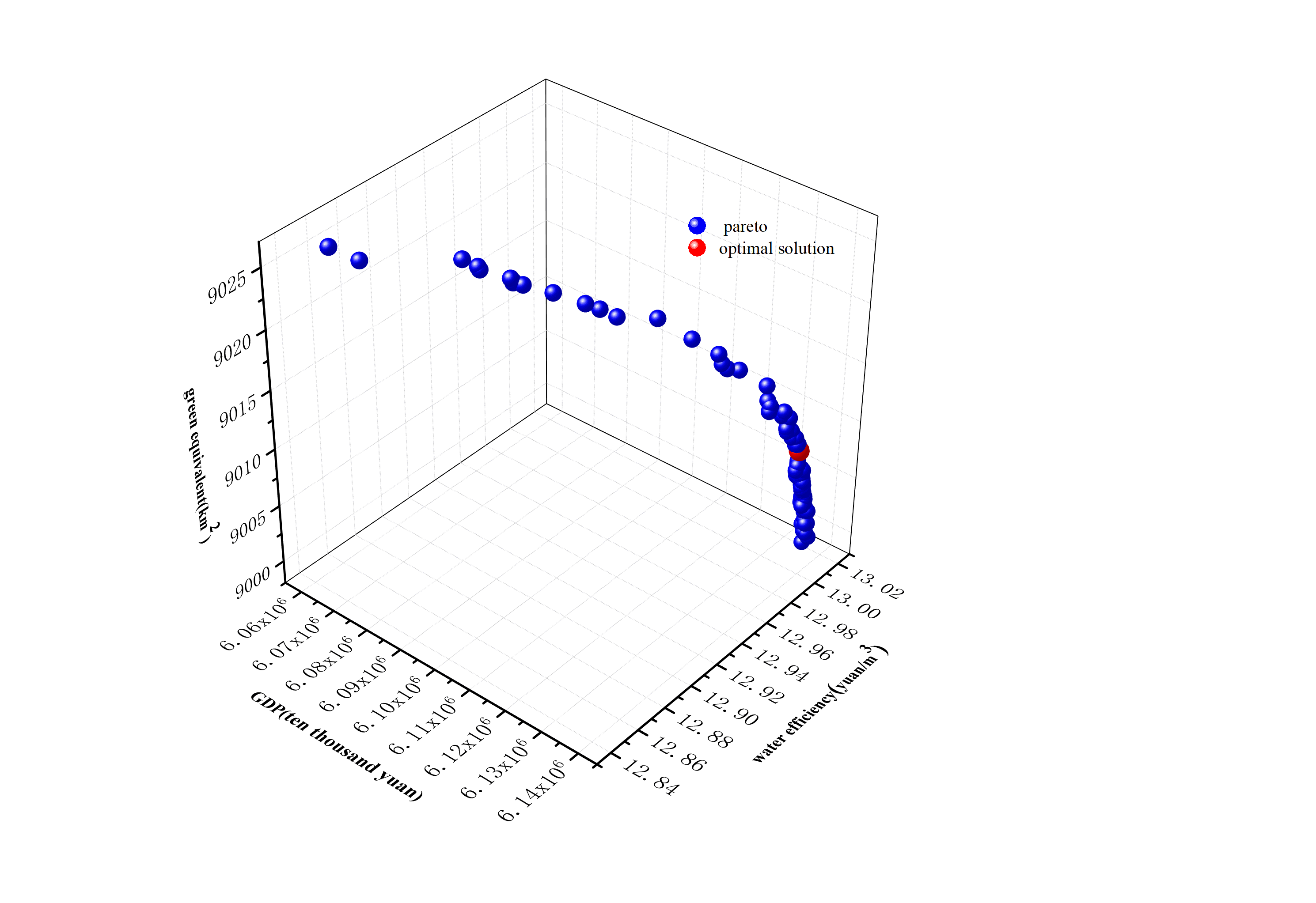 | |
| --- | --- | --- |
|  | RCP4.5 | RCP8.5 |
| 2035 | 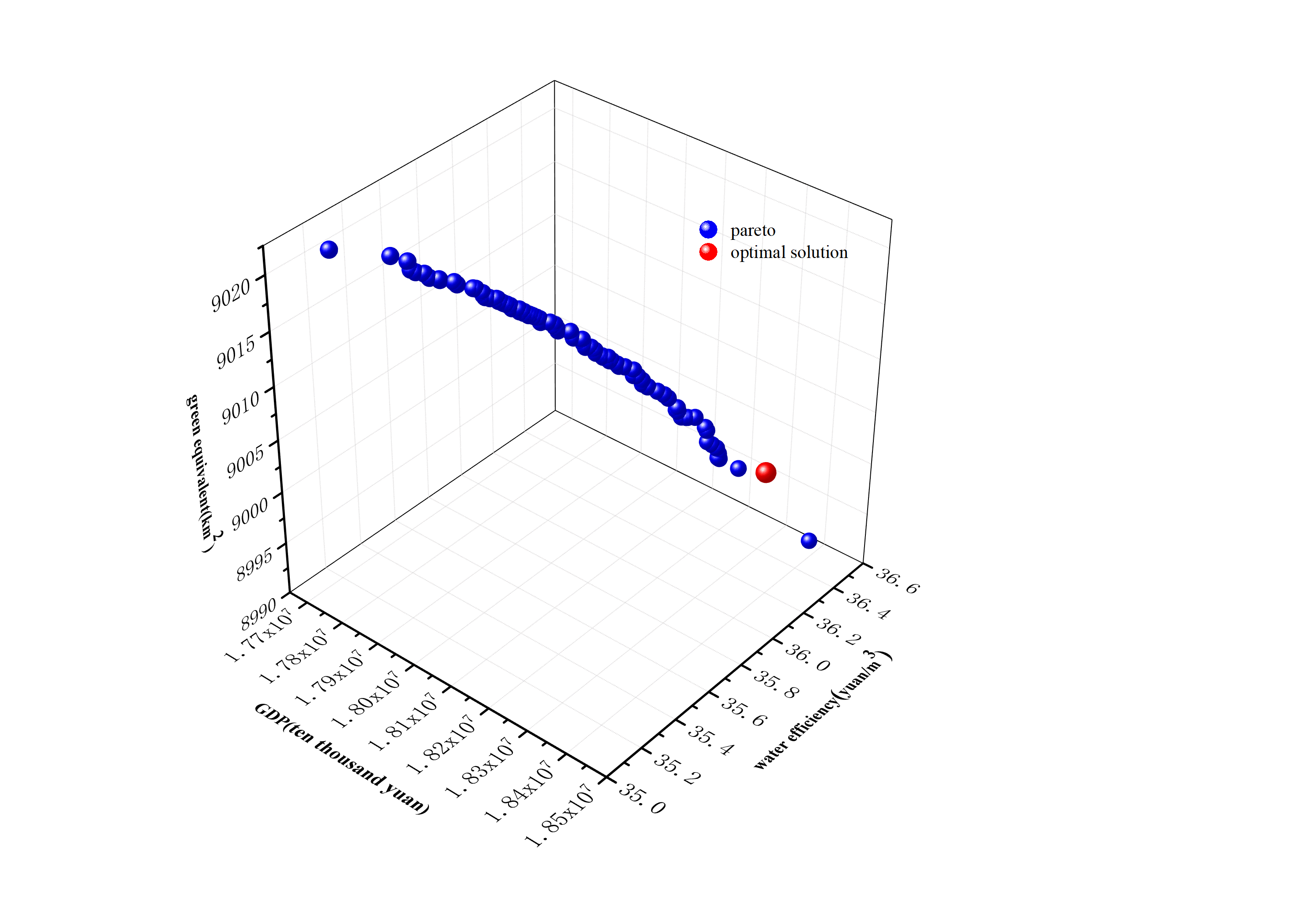 | 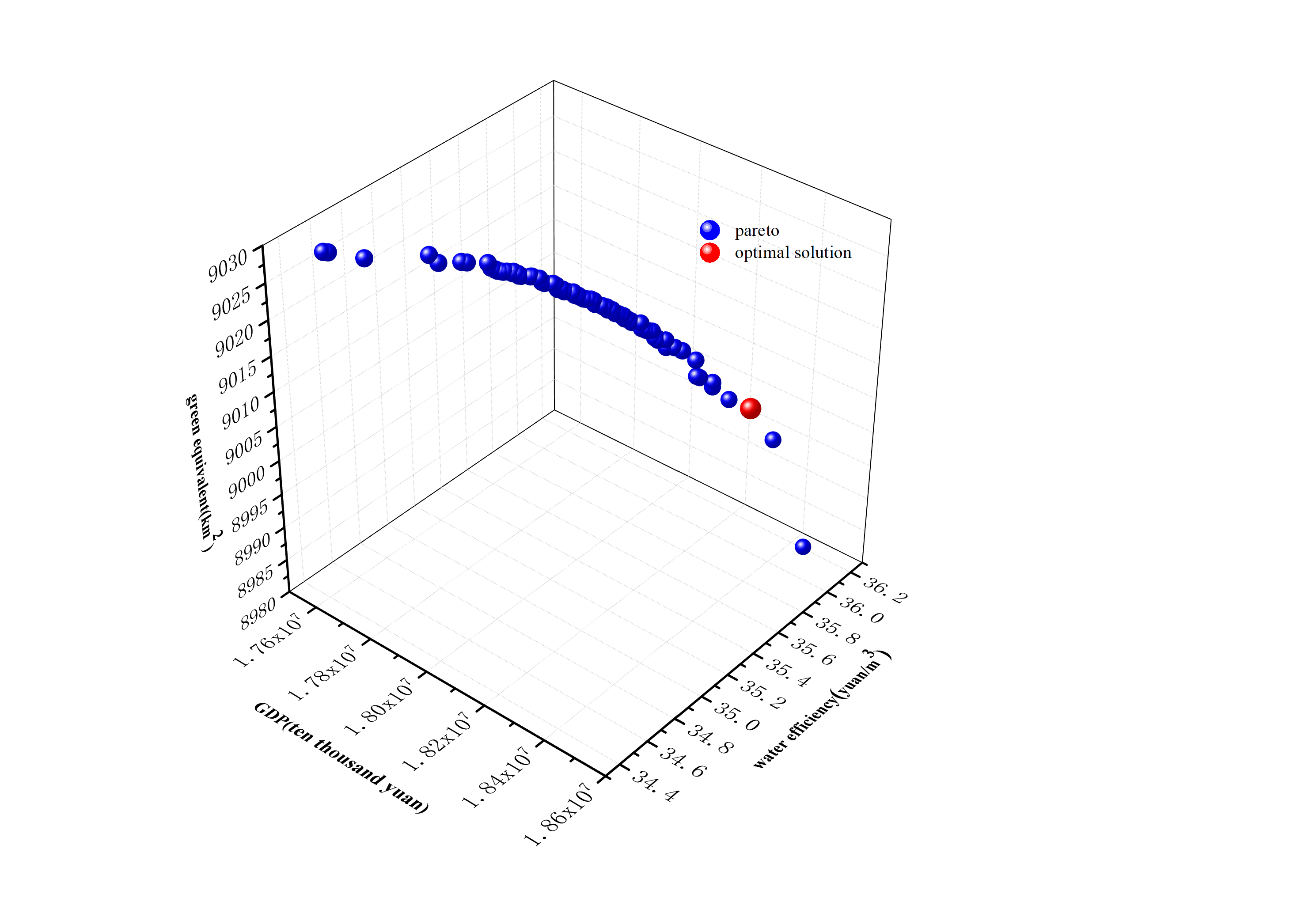 |
| 2050 | 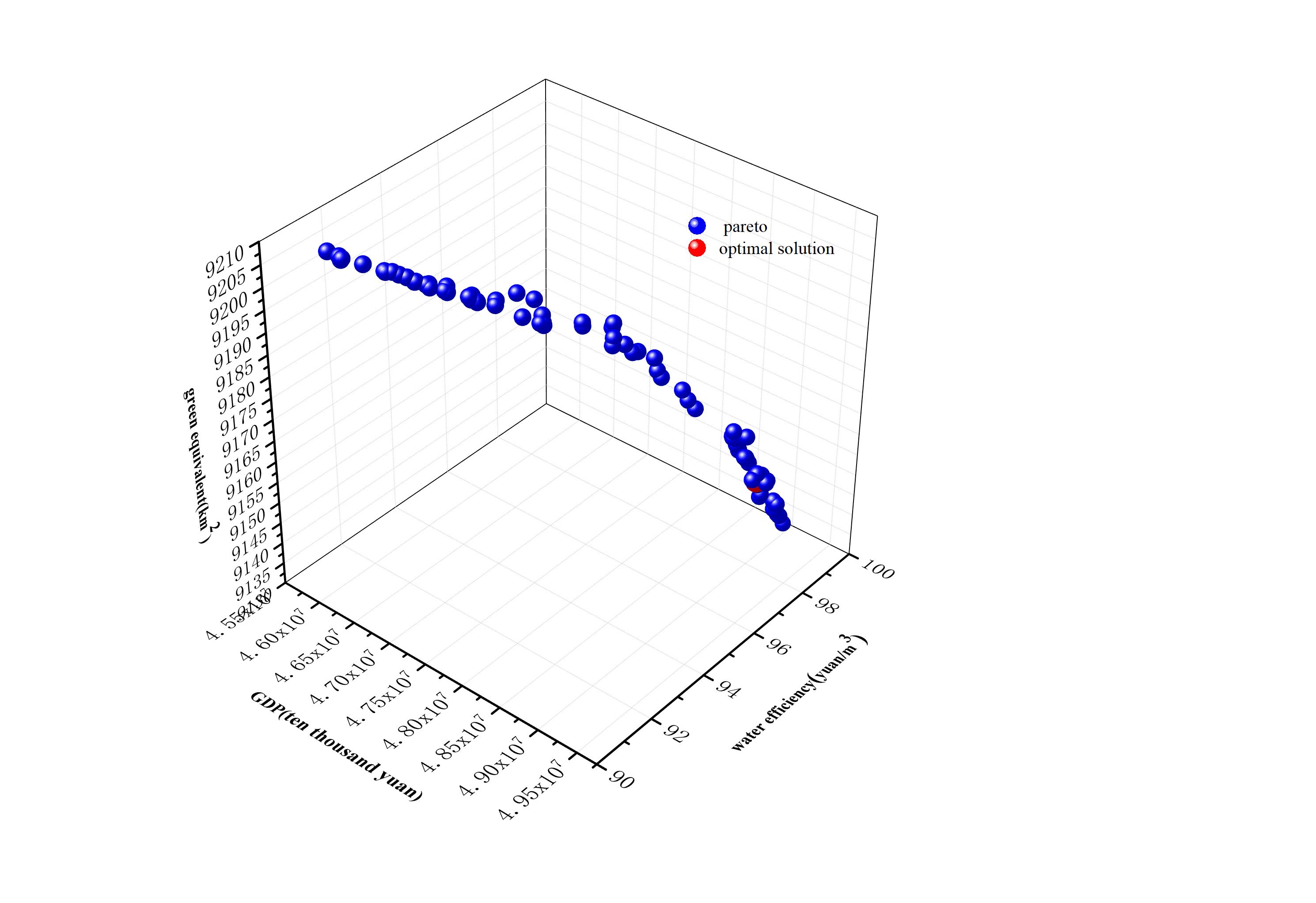 | 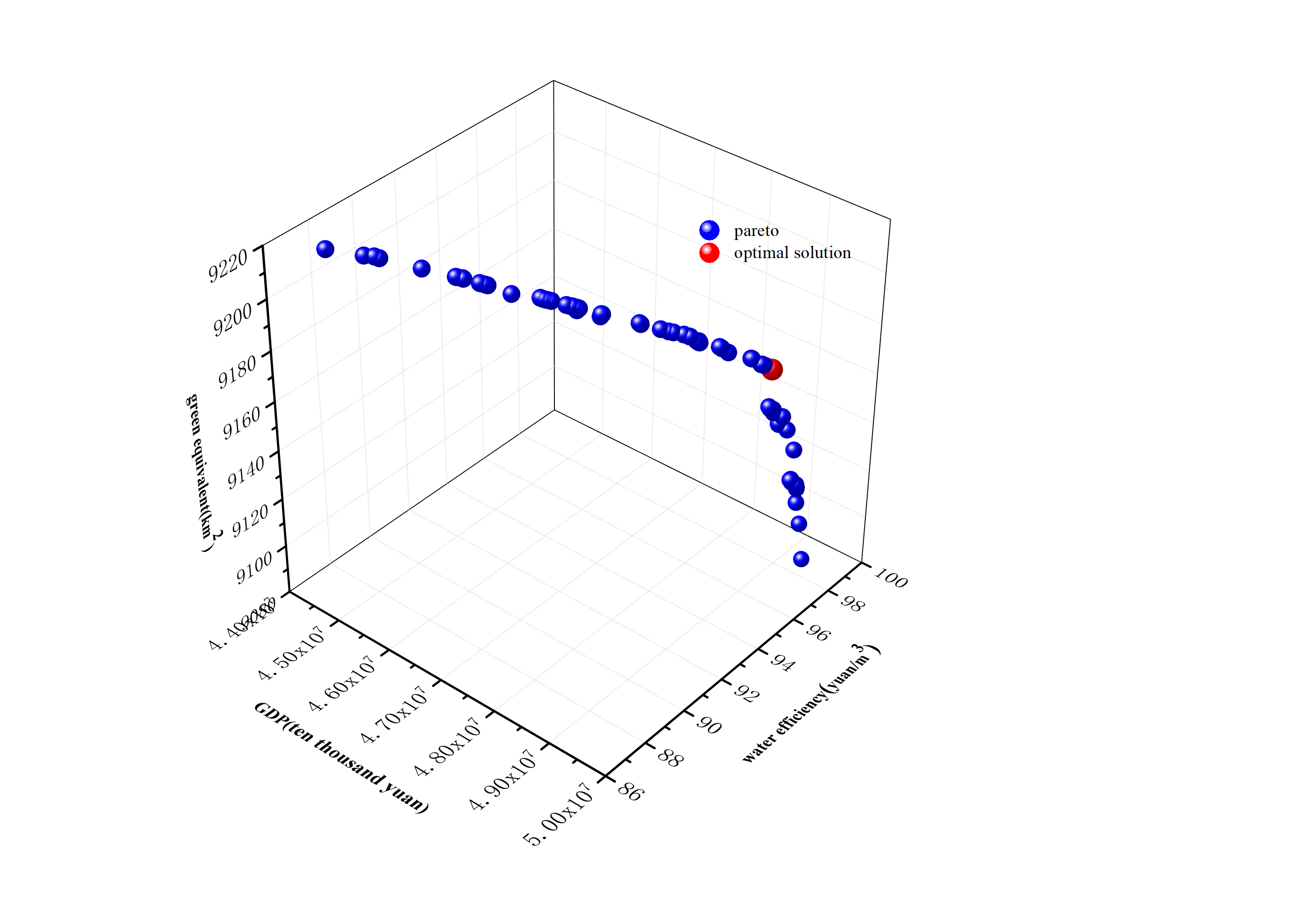 |

Figure 1 Pareto solution sets.
